# Cerebral Amyloid Angiopathy and the Risk of Hematoma Expansion

**DOI:** 10.1002/ana.26481

**Published:** 2022-08-27

**Authors:** David J. Seiffge, Alexandros A. Polymeris, Zhe Kang Law, Kailash Krishnan, Annaelle Zietz, Sebastian Thilemann, David Werring, Rustam Al‐Shahi Salman, Robert A. Dineen, Stefan T. Engelter, Philip M. Bath, Nikola Sprigg, Philippe Lyrer, Nils Peters, for the TICH‐2 Investigators

**Affiliations:** ^1^ Department of Neurology Inselspital University Hospital and University of Bern Bern Switzerland; ^2^ Department of Neurology and Stroke Center University Hospital Basel and University of Basel Basel Switzerland; ^3^ Stroke Trials Unit University of Nottingham Nottingham UK; ^4^ Department of Medicine National University of Malaysia Bangi Malaysia; ^5^ Stroke, Nottingham University Hospitals National Health Service Trust Nottingham UK; ^6^ Stroke Research Centre University College London Queen Square Institute of Neurology London UK; ^7^ Centre for Clinical Brain Sciences University of Edinburgh Edinburgh UK; ^8^ Radiological Sciences University of Nottingham Nottingham UK; ^9^ National Institute for Health Research Nottingham Biomedical Research Centre Nottingham UK; ^10^ Neurology and Neurorehabilitation, University Hospital for Geriatric Medicine Felix Platter University of Basel Basel Switzerland; ^11^ Stroke Center, Hirslanden Clinic Zürich Switzerland

## Abstract

**Objective:**

We assessed whether hematoma expansion (HE) and favorable outcome differ according to type of intracerebral hemorrhage (ICH).

**Methods:**

Among participants with ICH enrolled in the TICH‐2 (Tranexamic Acid for Hyperacute Primary Intracerebral Haemorrhage) trial, we assessed baseline scans for hematoma location and presence of cerebral amyloid angiopathy (CAA) using computed tomography (CT, simplified Edinburgh criteria) and magnetic resonance imaging (MRI; Boston criteria) and categorized ICH as lobar CAA, lobar non‐CAA, and nonlobar. The main outcomes were HE and favorable functional outcome. We constructed multivariate regression models and assessed treatment effects using interaction terms.

**Results:**

A total of 2,298 out of 2,325 participants were included with available CT (98.8%; median age = 71 years, interquartile range = 60‐80 years; 1,014 female). Additional MRI was available in 219 patients (9.5%). Overall, 1,637 participants (71.2%) had nonlobar ICH; the remaining 661 participants (28.8%) had lobar ICH, of whom 202 patients had lobar CAA‐ICH (8.8%, 173 participants according to Edinburgh and 29 participants according to Boston criteria) and 459 did not (lobar non‐CAA, 20.0%). For HE, we found a significant interaction of lobar CAA ICH with time from onset to randomization (increasing risk with time, *p*_interaction_ < 0.001) and baseline ICH volume (constant risk regardless of volume, *p*_interaction_ < 0.001) but no association between type of ICH and risk of HE or favorable outcome. Tranexamic acid significantly reduced the risk of HE (adjusted odds ratio = 0.7, 95% confidence interval = 0.6–1.0, *p* = 0.020) without statistically significant interaction with type of ICH (*p*_interaction_ = 0.058). Tranexamic acid was not associated with favorable outcome.

**Interpretation:**

Risk of HE in patients with lobar CAA‐ICH was not independently increased but seems to have different dynamics compared to other types of ICH. The time window for treatment of CAA‐ICH to prevent HE may be longer. ANN NEUROL 2022;92:921–930

Approximately 80% of nontraumatic intracerebral hemorrhage (ICH) is caused by bleeding‐prone cerebral small vessel disease (SVD),^1^ and this percentage could be even higher in older patients. Bleeding‐prone SVD comprises different subtypes of arteriopathy.^2^ Deep perforator arteriolopathy, mostly due to arterial hypertension, typically causes nonlobar ICH. Cerebral amyloid angiopathy (CAA) causes lobar ICH, but not all lobar ICH is due to CAA. In vivo diagnosis of CAA can be done using magnetic resonance imaging (MRI) and the modified Boston criteria,^3^ but this option is often limited by availability and/or feasibility of MRI. Recently, computed tomography (CT)‐based criteria to diagnose CAA have been developed (Edinburgh CAA criteria),^4^ overcoming limitations related to the use of MRI. These criteria are based on the presence of 2 distinct radiological markers (in addition to APOE genotype): fingerlike projections (FLP) and subarachnoid extension (SAH) of the hematoma. In the absence of information on APOE genotype, the combination of SAH and FLP can be used as a simplified version of the criteria.^5^

Hematoma expansion (HE) is a major determinant of poor outcome^6^ after ICH, and its prevention is the primary target of nonsurgical acute treatment approaches.^7^ Tranexamic acid, an antifibrinolytic drug, is effective in several bleeding‐related conditions such as severe trauma,^8^ by restricting bleeding. Tranexamic Acid for Hyperacute Primary Intracerebral Haemorrhage (TICH‐2) was a randomized controlled trial comparing the effectivity and safety of tranexamic acid compared to placebo in participants with nontraumatic ICH within 8 hours of onset.^9^ Participants with known macrovascular cause, oral anticoagulants, or other determined causes of ICH were excluded from TICH‐2; thus, the study population consisted virtually exclusively of participants with small vessel disease‐caused ICH.^10^

Knowledge about HE in participants with intracerebral hemorrhage due to different types of ICH is scarce,^11^ and the effect of tranexamic acid may vary depending on the underlying arteriopathy. The aim of this study was to assess the prevalence of CAA (defined using the CT‐based simplified Edinburgh CAA criteria or MRI‐based modified Boston criteria) and other types of ICH (ie, nonlobar and lobar non‐CAA ICH) among participants enrolled in the TICH‐2 trial, determine the association with HE and functional outcome, and assess the interaction of type of ICH with baseline hematoma volume, time since symptom onset, tranexamic acid treatment, and outcomes.

## Patients and Methods

We performed a post hoc analysis of the TICH‐2 trial. Details on participant characteristics, recruitment, procedures, and outcomes have been published previously.^9^, ^12^

### Study Population

The trial protocol^10^ and the results of the main analysis^9^ along with several post hoc analyses^13^, ^14^, ^15^, ^16^, ^17^, ^18^ have been published recently. TICH‐2 enrolled 2,325 participants with spontaneous, nontraumatic ICH within 8 hours of symptom onset. The trial excluded participants with macrovascular bleeding cause, prior anticoagulation, or other structural causes of hemorrhage (ie, tumor, thrombosis). For this post hoc analysis, we included all participants with baseline CT imaging available for central blinded analysis. We excluded participants with missing baseline CT imaging or if imaging was of poor quality, preventing assessment of FLP and SAH.

### Clinical Data Collection

Clinical data on demographics, clinical presentation on admission (National Institutes of Health Stroke Scale [NIHSS] and Glasgow Coma Scale, blood pressure), risk factors, and concomitant medication were collected by local investigators using a web‐based electronic databank with prespecified variables at baseline and during hospitalization. All participants received standardized follow‐up at 3 months to assess functional outcome using the modified Rankin Scale (mRS) score.

### Neuroimaging Analysis

All source CT images were coded and stored at the imaging core laboratory at the University of Nottingham, UK. Baseline and 24‐hour hematoma volume were measured using semiautomated volumetric analysis assessed by blinded raters for the purpose of the main trial. Intraventricular hemorrhage (IVH) was determined on baseline and follow‐up imaging. Hematoma location was classified as in the main trial and dichotomized into lobar (supratentorial hematoma located in the cerebral lobes) and nonlobar (deep and infratentorial).

For this analysis, 2 experienced vascular neurologists (D.J.S. and N.P.) assessed coded baseline CT scans for the presence of FLP and SAH as previously described.^4^ We assessed inter‐rater reliability in a subset of 75 randomly selected participants. In case of disagreement, an experienced neuroradiologist (R.A.D.) provided additional adjudication. All raters were blinded to outcome measures and TICH‐2 treatment allocation. We assessed available source images from the TICH‐2 MRI substudy^19^ for possible or probable CAA according to the modified Boston criteria.^3^

### Type of ICH

For this analysis, we classified participants into 1 of 3 three groups according to hematoma location and presence of CT markers of CAA (FLP and SAH) or probable CAA according to modified Boston criteria on MRI: nonlobar ICH, lobar non‐CAA ICH, and lobar CAA‐ICH. Participants with lobar hematoma location and simultaneous presence of both FLP and SAH (high probability of CAA according to Edinburgh CAA criteria) or fulfilling modified Boston criteria for probable CAA were classified as lobar CAA ICH.^4^ Participants with lobar hematoma location and presence of only 1 CT marker (either FLP or SAH) or none were classified as lobar non‐CAA ICH. All participants with a nonlobar hematoma location were classified as nonlobar ICH.

### Outcomes

The primary outcomes of this analysis were HE and favorable functional outcome (mRS = 0–2). HE was defined as an increase of hematoma volume of +33% or 6ml between baseline and follow‐up CT at 24 hours (defined as in the main TICH‐2 analysis). For sensitivity analysis, we used a combined definition of HE including new IVH on follow‐up imaging as recently published^20^ (ie, +33% or 6ml intraparenchymal HE or any new IVH on follow‐up imaging). Favorable functional outcome was defined as an mRS score of 0–2 at 3 months. Unfavorable outcome was defined as mRS = 3–6.

### Ethics

All participants or their next of kin/legal representative provided written informed consent for participation in TICH‐2.^9^ Ethics approval was obtained at each site and country before the start of the study. The trial is registered with the ISRCTN registry (ISRCTN93732214).

### Statistical Analysis

We stratified participants' characteristics by type of ICH (nonlobar, lobar non‐CAA, lobar CAA). We present categorical data using frequencies and percentages and continuous data using the median and interquartile range (IQR). To determine the association between outcomes (HE and favorable functional outcome) with type of ICH and Edinburgh CT markers, we constructed multivariate binary logistic regression models adjusting for covariates, as prespecified in the primary publication and statistical analysis plan. The following covariates were used: age, sex, systolic blood pressure on admission, NIHSS on admission, onset‐to‐randomization time, prior antiplatelet therapy, intraventricular hemorrhage on admission, and baseline hematoma volume. Post hoc, we added baseline blood glucose and prior treatment with statins to the multivariate models due to their known association with outcomes.

To test the interaction between type of ICH and treatment with tranexamic acid on outcomes, we introduced an interaction term between tranexamic acid and type of ICH in the aforementioned multivariate models, as well as interaction terms for type of ICH with time to randomization and hematoma volume, respectively, as both factors are known to influence HE and outcome. Results are presented as adjusted odds ratio (aOR) with 95% confidence interval (CI). We calculated predicted probability margins and plotted the results (marginsplot) for interaction analyses. We performed a post hoc sensitivity analysis using a different cutoff for definition of favorable and unfavorable functional outcome in accordance with the sensitivity analysis of the main TICH‐2 trial,^9^ where mRS of 0 to 3 was considered favorable and 4 to 6 unfavorable. We performed an additional post hoc analysis using the combined definition of HE, including new IVH on follow‐up imaging.^20^ All statistical analysis were carried out using Stata (v16.0; StataCorp, College Station, TX).

## Results

Among 2,325 participants enrolled in the TICH‐2 study, 2,298 participants were included in this analysis (98.8%). We excluded 27 participants due to missing baseline CT scan or insufficient imaging quality. In addition, 219 patients had additional MRI available (9.5%; 74 patients with lobar ICH = 11% of lobar ICH). Median age was 71 years (IQR = 60–80 years), 1,014 participants were female (44.1%), median baseline hematoma volume was 13.2cm^3^ (IQR = 5.5–32.2cm^3^), and median baseline NIHSS was 12 points (IQR = 7–19). Baseline characteristics are displayed in Table 1.

**TABLE 1:** Baseline Characteristics

Characteristic	All participants, N = 2,298	Nonlobar ICH, n = 1,637	Lobar non‐CAA ICH, n = 459	Lobar CAA ICH, n = 202	*p*
Demographics					
Age, yr	71 (60–80)	67 (56–78)	74 (66–82)	78 (72–84)	<0.001
Female sex	1,014 (44.1%)	633 (38.7%9	270 (55.6%)	121 (59.9%)	<0.001
Blood pressure on admission, mmHg					
Systolic	174 (154–193)	177 (158–196)	167 (150–190)	160 (143–185)	<0.001
Diastolic	93 (80–107)	96 (83–110)	88 (78–100)	83 (73–98)	<0.001
Blood pressure at 24 h, mmHg					
Systolic	149 (135–163)	150 (136–165)	143 (130–160)	145 (130–160)	<0.001
Diastolic	80 (70–90)	80 (70–91)	77 (68–86)	73 (66–84)	<0.001
Onset to randomization, h	3.6 (2.6–5.0)	3.5 (2.5–4.9)	3.9 (2.9–5.5)	3.7 (2.7–5.1)	<0.001
GCS on admission	15 (12–15)	15 (12–15)	15 (12–15)	14 (11–15)	<0.001
NIHSS on admission	12 (7–19)	12 (7–18)	11 (6–18)	17 (10–22)	<0.001
ICH volume at baseline in ml	13.2 (5.5–32.3)	9.6 (4.4–19.3)	27.5 (10.9–54.8)	63.7 (38.9–87.3)	<0.001
Intraventricular hemorrhage at baseline	745 (34.2%)	560 (34.2%)	118 (24.3%)	67 (33.2%)	<0.001
Prior antiplatelet agents	605 (26.4%)	422 (25.8%)	123 (26.8%)	60 (29.3%)	0.467
Prior statin therapy	615 (27.0%)	412 (25.4%)	135 (29.7%)	68 (33.8%)	0.014
Medical history					
Ischemic stroke or TIA	324 (14.3%)	217 (13.4%)	73 (16.0%)	34 (17.0%)	0.175
Hemorrhagic stroke	125 (5.5%)	58 (3.6%)	39 (8.6%)	28 (13.9%)	<0.001
Ischemic heart disease	199 (8.8%)	144 (8.9%)	34 (7.5%)	21 (10.6%)	0.400
Hypertension	1,406 (61.6%)	1,052 (64.6%)	252 (55.8%)	102 (50.5%)	<0.001
Diabetes mellitus	309 (13.5%)	251 (15.4%)	45 (9.9%)	13 (6.4%)	<0.001
Atrial fibrillation	70 (3.1%)	52 (3.2%)	10 (2.2%)	8 (4.0%)	0.373
Hyperlipidemia	587 (25.8%)	398 (24.5%)	126 (27.8%)	63 (31.3%)	0.063
Tranexamic acid	1,148 (50.0%)	816 (49.9%)	230 (50.1%)	102 (50.5%)	0.983

Continuous data are presented as median (interquartile range), categorical data as n (%).

CAA = cerebral amyloid angiopathy; GCS = Glasgow Coma Scale; ICH = intracerebral hemorrhage; NIHSS = National Institutes of Health Stroke Scale; TIA = transient ischemic attack.

There were 1,637 participants (71.2%) with nonlobar hematoma location (nonlobar ICH). Among the 661 participants with lobar hematoma location, 194 participants had FLP (8.4% of all participants and 29.4% of participants with lobar ICH) and 365 participants had SAH (15.9% of all ICH and 55.2% of lobar ICH). Inter‐rater reliability was moderate for FLP (80% agreement, Cohen kappa = 0.58) and substantial for SAH (88% agreement, Cohen kappa = 0.70).

Of the 661 participants with lobar hematoma location, 202 patients had lobar CAA ICH (30.6% of participants with lobar hematoma and 8.8% of all participants with ICH; 173 fulfilled CT‐based Edinburgh criteria for high probability of CAA, and 29 additional patients fulfilled MRI‐based modified Boston criteria for probable CAA) and the remaining 459 had non‐CAA ICH (ie, fulfilling neither CT‐based Edinburgh criteria for high probability nor MRI‐based modified Boston criteria for probable CAA; 69.4% of participants with lobar ICH and 20.0% of all participants with ICH). Of these 459 participants, 199 participants had 1 CT marker (181 participants had SAH and 18 had FLP). Treatment allocation to tranexamic acid was equally distributed among all subtypes of ICH (49.9% nonlobar ICH, 50.1% lobar non‐CAA ICH, and 50.5% lobar CAA‐ICH). Median hematoma volume was 9.6cm^3^ (IQR = 4.4–19.3) in participants with nonlobar ICH, 27.5cm^3^ (IQR = 10.9–54.8) in participants with lobar non‐CAA ICH, and 63.7cm^3^ (IQR = 38.9–87.3) in participants with lobar CAA‐ICH.

### Hematoma Expansion

**FIGURE 1:**
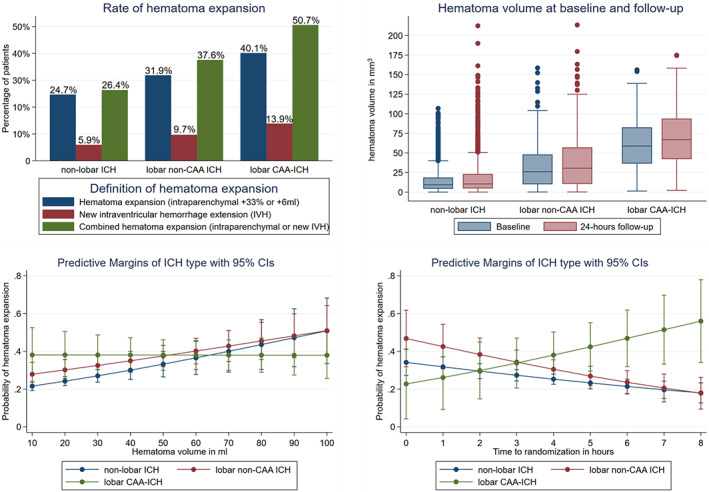
Risk of hematoma expansion (HE) according to type of intracerebral hemorrhage (ICH). Percentage of participants with HE (≥33% or ≥6ml; upper left panel) and absolute hematoma volume at baseline and 24‐hour follow‐up computed tomography (upper right panel). There was a significant interaction on the risk of HE of type of ICH with hematoma volume (lower left panel, in contrast to other types of ICH, the risk of HE seems constant for lobar cerebral amyloid angiopathy [CAA]‐ICH regardless of baseline volume, *p*_interaction_ < 0.001) and time to randomization (lower right panel, increasing risk with time in participants with lobar CAA‐ICH, *p*_interaction_ <0.001). Predicted probability was assessed using multivariate analysis adjusting for covariates (age, sex, National Institutes of Health Stroke Scale at baseline, systolic blood pressure at baseline, intraventricular hemorrhage [IVH] at baseline, prior antiplatelet therapy, time from onset to randomization, baseline blood glucose, prior statin therapy, and baseline ICH volume) and interaction terms for time and volume with type of ICH. CI = confidence interval. [Color figure can be viewed at www.annalsofneurology.org]

**FIGURE 2:**
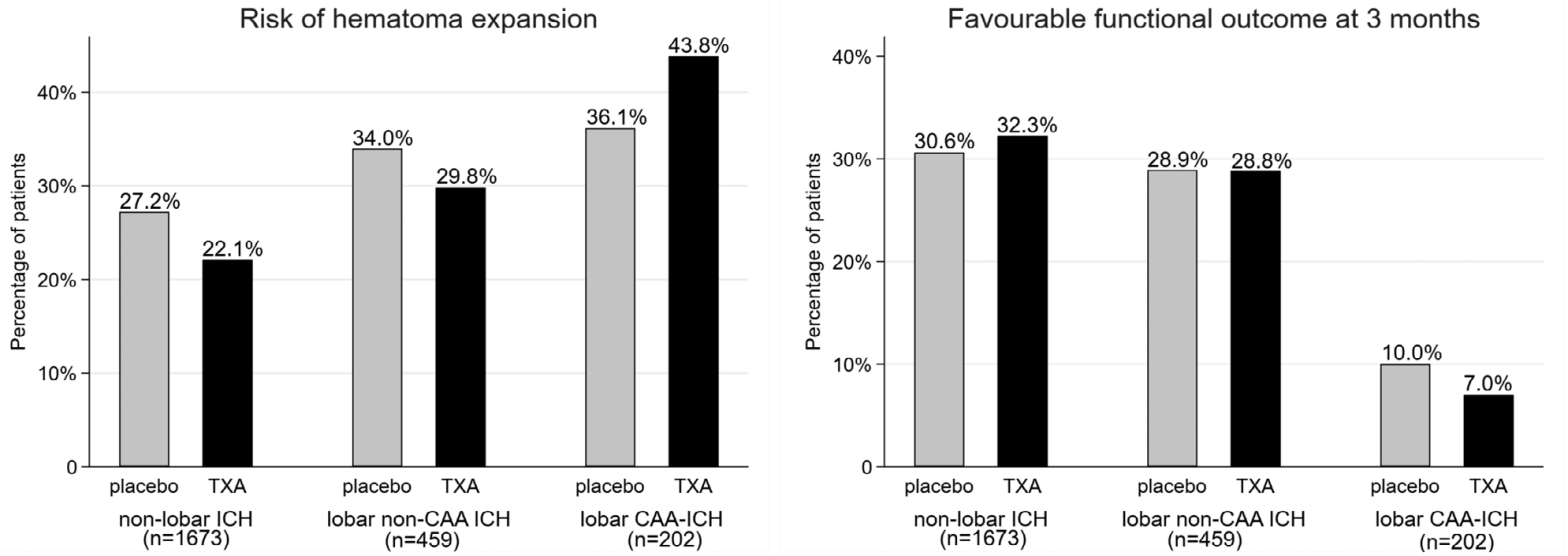
Risk of hematoma expansion (left) and chance of good functional outcome (modified Rankin Scale = 0–2 at 3 months; right) according to type of intracerebral hemorrhage (ICH) in participants receiving placebo or tranexamic acid (TXA). We did not observe any statistically significant interaction between treatment with tranexamic acid and type of ICH, either on risk of hematoma expansion (*p*_interaction_ = 0.214) or on good functional outcome (*p*_interaction_ = 0.710). CAA = cerebral amyloid angiopathy.

Overall, 562 participants had HE (27.4%) and 172 participants (7.4%) had new IVH on follow‐up imaging, with 638 participants (30.7%) having combined HE (ie, HE or new IVH on follow‐up imaging; Fig 1). In multivariate analysis, SAH (OR = 1.7, 95% CI = 1.1–2.5, *p* = 0.014) but not FLP was associated with HE (Table 2). Rate of HE and absolute volumes at baseline and follow‐up imaging according to type of ICH are displayed in Figure 1. We found a significant interaction between type of ICH and time from onset to randomization (increasing risk of HE with time in participants with lobar CAA‐ICH, *p*_interaction_ < 0.001) and baseline ICH volume (in contrast to other types of ICH, the risk of HE seems constant for lobar CAA‐ICH regardless of baseline volume, *p*_interaction_ < 0.001) on HE. Compared to nonlobar ICH, both lobar non‐CAA ICH and lobar CAA‐ICH were associated with higher rates of HE in univariate analysis but not after adjusting for covariates (see Table 2). Tranexamic acid significantly reduced HE in all participants (placebo 301 patients with HE = 29.2% vs tranexamic acid 261 patients with HE = 25.4%, aOR = 0.7, 95% CI = 0.6–1.0, *p* = 0.020). Figure 2 displays risk of HE according to type of ICH and treatment with tranexamic acid or placebo and the predicted probability of HE according to type of ICH. Whereas the unadjusted rate of HE was lower with tranexamic acid compared to placebo in patients with nonlobar ICH and lobar non‐CAA ICH, this was not the case in patients with lobar CAA‐ICH (see Fig 2). There was no statistically significant interaction between treatment with tranexamic acid and type of ICH (*p*_interaction_ = 0.214) on HE. In post hoc analysis, using the combined definition of HE (+33%, 6ml intraparenchymal HE, or any new IVH) did not change the results (data not shown).

**TABLE 2:** Binary Regression Analysis for Hematoma Expansion and Favorable Functional Outcome (mRS = 0–2 at 3 Months)

	Univariate		Multivariate	
	Odds ratio (95% CI)	*p*	Odds ratio (95% CI)	*p*
Hematoma expansion				
CT imaging marker				
FLP	1.4 (1.0–2.1)	0.061	0.9 (0.6–1.5)	0.784
SAH	2.4 (1.6–3.2)	<0.001	1.7 (1.1–2.5)	0.014
Type of ICH				
Nonlobar ICH	1 [reference]	1 [reference]	1 [reference]	1 [reference]
Lobar non‐CAA ICH	1.7 (1.3–2.1)	<0.001	1.7 (0.8–3.6)	0.184
Lobar CAA‐ICH	2.9 (2.1–3.9)	<0.001	0.8 (0.2–2.8)	0.709
Favorable functional outcome				
CT imaging marker				
FLP	0.1 (0.1–0.3)	<0.001	0.5 (0.2–1.2)	0.109
SAH	0.2 (0.1–0.3)	<0.001	0.8 (0.5–1.4)	0.468
Type of ICH				
Nonlobar ICH	1 [reference]	1 [reference]	1 [reference]	1 [reference]
Lobar non‐CAA ICH	0.9 (0.7–1.2)	0.281	1.0 (0.4–2.3)	0.934
Lobar CAA‐ICH	0.2 (0.1–0.3)	<0.001	0.1 (0.0–2.0)	0.147

Multivariate analysis was adjusted for age, sex, National Institutes of Health Stroke Scale at baseline, systolic blood pressure at baseline, intraventricular hemorrhage at baseline, prior antiplatelet therapy, time from onset to randomization, baseline blood glucose, prior statin therapy, and baseline ICH volume, including interaction terms for type if ICH with time to randomization and baseline ICH volume. CAA = cerebral amyloid angiopathy; CI = confidence interval; CT = computed tomography; FLP = fingerlike projections; ICH = intracerebral hemorrhage; mRS = modified Rankin Scale; SAH = subarachnoid extension.

### Functional Outcome

**FIGURE 3:**
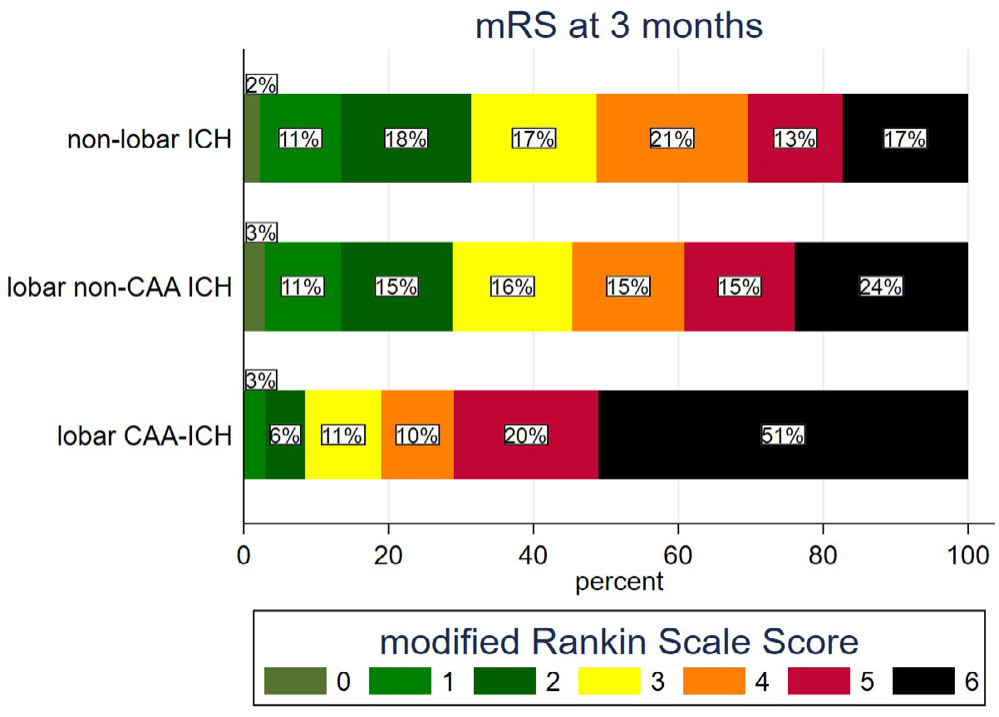
Distribution of modified Rankin Scale (mRS) scores at 3 months per type of intracerebral hemorrhage (ICH). CAA = cerebral amyloid angiopathy. [Color figure can be viewed at www.annalsofneurology.org]

**FIGURE 4:**
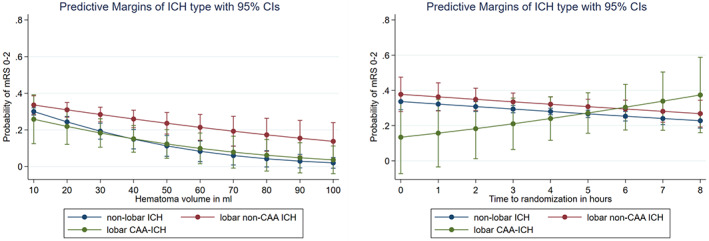
There was no statistically significant interaction between type of intracerebral hemorrhage (ICH) and hematoma volume (left panel; *p*_interaction_ = 0.214) or time to randomization (right panel; *p*_interaction_ = 0.359) on chance of favorable outcome (modified Rankin Scale [mRS] = 0–2 at 3 months). Predicted probability was assessed using multivariate analysis adjusting for covariates (age, sex, National Institutes of Health Stroke Scale at baseline, systolic blood pressure at baseline, intraventricular hemorrhage at baseline, prior antiplatelet therapy, time from onset to randomization, prior statin therapy, baseline blood glucose, and baseline ICH volume) and interaction terms for time and volume with type of ICH. CAA = cerebral amyloid angiopathy; CI = confidence interval. [Color figure can be viewed at www.annalsofneurology.org]

Overall, 663 participants (29.1%) had favorable outcome (mRS = 0–2 at 3 months) and 1,239 (54.3%) had poor outcome (mRS = 4–6 at 3 months, sensitivity analysis). Neither SAH nor FLP were independently associated with favorable outcome. Distribution of mRS at 3 months according to type of ICH is displayed in Figure 3. There was no statistically significant interaction between type of ICH and time from onset to randomization (*p*_interaction_ = 0.203) or baseline ICH volume (*p*_interaction_ = 0.201) on chance of favorable outcome (Fig 4). Lobar CAA‐ICH but not lobar non‐CAA ICH was associated with reduced odds of favorable outcome in univariate analysis (see Table 2). After adjusting for covariates, we found no significant association between type of ICH and favorable outcome.

Figure 4 displays distribution of favorable outcome at 3 months according to type of ICH and treatment with tranexamic acid or placebo. Tranexamic acid was not associated with favorable outcome (aOR = 1.3, 95% CI = 1.0–1.7, *p* = 0.098), and there was no significant interaction with type of ICH (*p*_interaction_ = 0.313). In the sensitivity analysis, there was no association between type of ICH and poor outcome (mRS = 4–6) and no interaction with time, volume, or treatment with tranexamic acid.

## Discussion

This post hoc analysis of individual participant data from the randomized TICH‐2 trial revealed the following findings. (1) Among participants enrolled in TICH‐2, the majority of patients had nonlobar ICH and only 8.8% had CAA as defined by CT (simplified Edinburgh criteria) or MRI (modified Boston criteria). (2) HE was frequent and functional outcome was poor among participants with lobar CAA‐ICH, but there was no statistically significant association between type of ICH and outcomes. (3) In patients with lobar CAA‐ICH, there was a significant interaction between time from randomization (increasing with time) and baseline hematoma volume (constant regardless of baseline volume) and risk of HE. (4) Tranexamic acid significantly reduced HE. Although this finding was driven by reduced HE in participants with nonlobar and lobar non‐CAA ICH, there was no statistically significant interaction with type of ICH. There was no effect of tranexamic acid on favorable outcome and no evidence for interaction with type of ICH.

Previous studies provided insights into HE according to hematoma location, dichotomizing lobar and nonlobar ICH without further differentiating potential underlying types of arteriolopathy.^21^, ^22^, ^23^, ^24^ However, lobar ICH consist of heterogeneous pathologies, including CAA‐related ICH but also hypertension‐associated deep perforator arteriolopathy and mixed SVD.^4^ Only 58% of participants with lobar ICH have CAA pathology on postmortem autopsy studies, with the majority of participants having mixed SVD and only 16% having “pure” CAA pathology without any other SVD.^4^ One prior report suggested differences in HE rates among different subtypes of ICH, but generalizability was limited due to small sample size and a single center retrospective design.^11^ A different study using genetic data found increased rates of HE in participants with lobar hematoma location and APOE e2 genotype (associated with CAA).^25^ We applied CT‐based and pathologically validated criteria for CAA^4^ (Edinburgh criteria), which allowed probable diagnosis of CAA in a broad spectrum of participants with ICH not limited by availability or feasibility of MRI^3^ or genetic testing. The findings of our study provide novel insights about HE in participants with different types of ICH. Although no specific type of ICH was associated with increased risk of HE, there seems to be a significant interaction between type of ICH, time since onset, and baseline hematoma volume on HE in patients with lobar CAA‐ICH, which is remarkably different from those with other types of ICH. This finding goes against the “avalanche” theory of HE, where bleeding starts from one primary vessel and as the hematoma grows, it sheers the surrounding secondary vessels, causing more bleeding.^26^ The pathophysiological rationale behind the difference observed in our study remains unclear. We can only hypothesize that CAA‐related bleeding originates from leptomeningeal vessels and CAA usually affects a significant proportion of these leptomeningeal arterioles in the brain of patients with CAA‐associated ICH.^27^ The architecture of leptomeningeal and pial vessels forms an effective collateral network,^28^ as opposed to deep penetrating arterioles that are end arteries. In this setting, vasoconstriction response of hemostasis may be less effective when there are collaterals. In addition, the lobar location creates more space (ie, in the subarachnoid space, within the sulci) and less chance of tamponade stopping the bleeding compared to nonlobar location. Taken together, these mechanisms may result in constant and prolonged bleeding and HE occurring even after hours and regardless of baseline hematoma volume in patients with lobar CAA‐ICH. This prolonged risk of HE may actually mean that these patients have a potentially longer treatment window for hemostatic therapies, which needs to be determined in further studies.

We furthermore assessed the efficacy of tranexamic acid among different types of ICH, closing an existing gap of knowledge. Our data suggest that treatment with tranexamic acid reduces the risk of HE in all participants, and type of ICH did not modify treatment effects. This effect seems to be driven by the effect of tranexamic acid on nonlobar hemorrhage. In participants with lobar CAA‐ICH, we found the predicted probability of HE to be higher in those who received tranexamic acid compared to those who received placebo, although the results were statistically not significant. We can therefore not rule out a potential neutral or even harmful effect of tranexamic acid in patients with lobar CAA‐ICH. One explanation for this finding is the interaction between CAA, the fibrinolytic system, and tranexamic acid, but current knowledge needs further exploration.^29^ We urge caution in the interpretation of these results due to the limited sample size of the lobar CAA‐ICH group.

We used the CT‐based Edinburgh criteria for CAA. Inter‐rater reliability for key imaging markers (FLP and SAH) was moderate to substantial and well in line with the original publication of these criteria.^4^ Although participants with CAA‐ICH in our study had larger hematoma volumes compared to nonlobar ICH and lobar non‐CAA ICH, our findings are in line with the derivation cohort for the Edinburgh CAA criteria.^4^ Together, these findings support feasibility and reproducibility of the criteria. However, due to the derivation cohort (postmortem), there seems to be the possibility that whereas these criteria primarily capture large CAA‐related ICH, smaller CAA‐related hematomas do not fulfil the criteria and remain underdiagnosed. A recent study found a good calibration for the simplified CT‐based Edinburgh criteria and the MRI‐based modified Boston criteria.^5^

Our study has several strengths. (1) We used data from a large and well‐characterized cohort included in a randomized controlled trial with excellent data quality and completeness. (2) All participants were enrolled within 8 hours of symptom onset within the crucial time window of HE. (3) All participants received standardized follow‐up CT scans to assess HE. (4) Using CT‐based diagnosis of CAA allowed for a broad inclusion of patients, overcoming restrictions of studies using MRI as the exclusive modality to diagnose CAA. Based on MRI‐criteria, we included additional patients with CAA, thus using a complementary, multimodal approach to diagnose CAA. (5) The sample size of patients with CAA‐ICH was rather large compared to previous studies.

Our study has some limitations. (1) Based on imaging performed in TICH‐2, we used a CT‐based classification for CAA, which may be prone to bias toward severe, large hematomas. (2) There is a potential selection bias related to trial inclusion criteria, for example, excluding participants with premorbid disability. (3) Additional MRI was available in only 9% of patients, limiting diagnosis of CAA using modalities other than CT. Ideally, in vivo diagnosis of CAA should include different complementing modalities (ie, CT, MRI, cerebrospinal fluid biomarkers, positron emission tomography scan, APOE phenotype, and brain biopsy), as no single modality offers 100% sensitivity to detect CAA. Future studies may include different and complementing investigations to diagnose CAA, capturing a maximum of potential cases related to CAA. (4) Blood pressure variability is a predictor of HE,^30^ but this information was not available in this study. (5) Although interaction analysis did not show a significant interaction between type of ICH and treatment with tranexamic acid, the unadjusted number of patients with HE was higher in patients with lobar CAA‐ICH receiving tranexamic acid compared to those receiving placebo. Due to the small sample size of the group of patients with lobar CAA‐ICH, the interaction analysis is potentially underpowered, and we urge caution in the interpretation of these results.

Taken together, our data suggest that the association of time and baseline hematoma volume with HE in patients with lobar CAA‐ICH differs from other types of ICH, a finding that needs external validation in other cohorts. We did not find a clear association of the type of ICH with outcome or a modification of the treatment effect of tranexamic acid by type of ICH. The relation between lobar CAA‐ICH and treatment response to tranexamic acid warrants further investigations. Further trials of hemostatic agents should assess treatment effects in different subgroups.

## Author Contributions

D.J.S., N.S., P.M.B., D.W., R.A.‐S.S., R.A.D., S.T.E., P.L., and N.P. contributed to the concept and design of the study. D.J.S., A.A.P., Z.K.L., K.K., A.Z., S.T., R.A.D., and N.P. contributed to acquisition and analysis of data. D.J.S. and N.P. contributed to drafting the text or preparing the figures.

## Potential Conflicts of Interest

Nothing to report.

## Data Availability

Requests for data should be addressed to the chief investigator Professor Nikola Sprigg for consideration and in line with legal obligations and data protection laws.
